# Disease patterns in high-cost individuals with multimorbidity: a retrospective cross-sectional study in primary care

**DOI:** 10.3399/BJGP.2023.0026

**Published:** 2024-02-06

**Authors:** Marina Soley-Bori, Mark Ashworth, Alice McGreevy, Yanzhong Wang, Stevo Durbaba, Hiten Dodhia, Julia Fox-Rushby

**Affiliations:** London borough of Lambeth;; London borough of Lambeth;; London borough of Lambeth;; London borough of Lambeth;; London borough of Lambeth;; London borough of Lambeth;; School of Life Course & Population Sciences, King’s College London, London.

**Keywords:** electronic health records, high cost, long-term conditions, multimorbidity, primary care

## Abstract

**Background:**

‘High-cost’ individuals with multimorbidity account for a disproportionately large share of healthcare costs and are at most risk of poor quality of care and health outcomes.

**Aim:**

To compare high-cost with lower-cost individuals with multimorbidity and assess whether these populations can be clustered based on similar disease patterns.

**Design and setting:**

A cross-sectional study based on 2019/2020 electronic medical records from adults registered to primary care practices (*n* = 41) in a London borough.

**Method:**

Multimorbidity is defined as having ≥2 long-term conditions (LTCs). Primary care costs reflected consultations, which were costed based on provider and consultation types. High cost was defined as the top 20% of individuals in the cost distribution. Descriptive analyses identified combinations of 32 LTCs and their contribution to costs. Latent class analysis explored clustering patterns.

**Results:**

Of 386 238 individuals, 101 498 (26%) had multimorbidity. The high-cost group (*n* = 20 304) incurred 53% of total costs and had 6833 unique disease combinations, about three times the diversity of the lower-cost group (*n* = 81 194). The trio of anxiety, chronic pain, and depression represented the highest share of costs (5%). High-cost individuals were best grouped into five clusters, but no cluster was dominated by a single LTC combination. In three of five clusters, mental health conditions were the most prevalent.

**Conclusion:**

High-cost individuals with multimorbidity have extensive heterogeneity in LTCs, with no single LTC combination dominating their primary care costs. The frequent presence of mental health conditions in this population supports the need to enhance coordination of mental and physical health care to improve outcomes and reduce costs.

## Introduction

Multimorbidity, the presence of ≥2 long-term conditions (LTCs), has become a major public health concern across healthcare systems.^1^^–^3 With an ageing population and higher prevalence of conditions, such as obesity in younger cohorts, multimorbidity is now the norm across Europe.^4^ In the UK, two-thirds of people aged >65 years are expected to live with multimorbidity by 2035.^5^ Primary care is organised into general practices, which are the first point of contact for health needs and play a major role in preventing, diagnosing, and caring for individuals with multimorbidity.^6^^–^8 Funding and recruitment shortfalls have resulted in increasing workload, with more patients per GP,^9^^,^^10^ challenging their ability to care properly for people with multimorbidity.^11^^,^^12^ This problem is magnified among ‘high-cost’ individuals with multimorbidity^13^ who have the highest risk of unmet health need, poorly coordinated and duplicated care, and worse health outcomes.^14^^,^^15^ Understanding the healthcare needs of these patients better can inform quality improvement and cost containment efforts.^16^

Little is known about high-cost individuals with multimorbidity. Research into the prevalence and combinations of LTCs has considered either the overall multimorbid population or separate age groups.^17^^–^20 Clusters of LTCs are defined by prevalence to date and may not correspond to the patient groups with worse outcomes and/or more frequent health service use.^21^ In a nationally representative sample from the UK, Zhu *et al* identified the cluster with a high prevalence of depression, anxiety, and pain as having the greatest health service use.^18^ Soley-Bori *et al* found that the disease/condition-based clusters of alcohol and substance dependence, followed by anxiety and depression, had the highest primary care use as additional LTCs develop over time.^22^ However, understanding the distribution and concentration of costs is needed,^13^ and identifying clusters based on primary care costs rather than disease prevalence may yield different results. International literature reports highly concentrated hospital costs, where the top 10% of patients account for between 50% and 80% of costs.^23^ Similar evidence in primary care, particularly within the multimorbid population in the UK, is lacking.

**Table:** How this fits in

‘High-cost’ individuals with multimorbidity tend to have the highest risk of unmet health need, poorly coordinated and duplicated care, and worse health outcomes. Understanding the healthcare needs of these individuals is essential to inform quality improvement and cost containment efforts. To the authors’ knowledge, this is the first study to characterise high-cost individuals with multimorbidity based on their primary care costs and disease prevalence, and compare them with lower-cost individuals. The most expensive combinations of diseases were identified and whether high-cost individuals can be clustered based on similar patterns of disease was assessed using a young, urban, multi-ethnic population-based sample.

This study aimed to:

- characterise and compare high-cost with lower-cost individuals with multimorbidity in primary care, in terms of LTC combinations and their contribution to primary care costs; and
- assess whether these populations can be grouped into clusters of similar LTCs.

## Method

### Study design, setting, participants, and data

This retrospective cross-sectional study of 386 238 electronic primary care medical records included adults (aged ≥18 years) registered between 1 April 2019 and 31 March 2020 at 41 general practices in the Lambeth DataNet. Individuals with multimorbidity (defined as the co-occurrence of ≥2 of 32 selected LTCs, Supplementary Box S1) totalled 101 498.

Patient-level primary care costs reflect primary care use and workload, measured by consultations. Consultations were costed for type of provider delivering care (GP, nurse, or other healthcare professional) and mode of delivery (face to face, telephone, home visits, or electronic). Other healthcare professionals included pharmacists, healthcare assistants/support workers, and physician associates. National unit costs from the year 2019–2020 were used for valuation, adjusted by average duration of consultation.^24^ For example, to cost a GP face-to-face consultation, the cost per hour for a GP (£255) was adjusted for the average duration of a GP face-to-face consultation (9.22 min), resulting in a unit cost of £39 (£255 × (9.22/60)).

High-cost individuals were defined as those at the top 20% (≥80th percentile) of the primary care cost distribution. Lower-cost individuals were the remaining sample (<80%). Although thresholds are often set at 10% or 5% quantiles,^14^^,^^25^ the current study’s more inclusive approach yields a larger sample size, still reflects higher-cost individuals, and allows assessment of the principle of cost disproportionality,^26^ where the relative medical expenditure of a population is larger than their relative size. Sensitivity analyses using the 70th and 90th percentile thresholds were conducted.

### Statistical methods

The full distribution of primary care costs are described for high-and lower-cost groups. Demographic characteristics (age, sex, ethnicity, and Index of Multiple Deprivation [IMD 2019]), death rates, prevalence of LTCs, consultations, and costs were summarised. Differences in these variables between high- and lower-cost individuals were tested using *t*-tests (Mann–Whitney *U*-test for non-normal distributions) and Pearson χ^2^ tests (for categorical variables). Missing data were kept as missing (Supplementary Box S2).

With 32 LTCs, the possible number of disease combinations is extensive (496[ = 16 × 31] pairs, 4960[ = 32!/(29!×3!]) triads, 35 960 (= 32!/[28!×4!]) tetrads, for example). To characterise disease heterogeneity among high- and lower-cost individuals, LTC combinations were identified with average and total costs computed. This step identified combinations of LTCs with a major contribution to primary care costs.

Latent class analysis (LCA) explored whether multimorbid individuals could be grouped into a more parsimonious and interpretable set of clusters based on their underlying LTC prevalence. Each patient cluster captures a different LTC prevalence and combination, with up to five classes tested. The final model was chosen by balancing goodness of fit measure, model stability, and convergence.^27^ Entropy then measured model fit.^28^ The resulting clusters were described based on demographic characteristics, death rates, average consultations and costs, average number of LTCs, and prevalence of individual LTCs and LTC combinations. Further details are in Supplementary Box S2.

SAS (version 9.4) was used for analyses, including PROC LCA to implement the clustering technique. This study is reported using STROBE guidelines (https://www.strobe-statement.org).

## Results

### Population

High-cost individuals with multimorbidity (*n* = 20 304) were older, more often female, from ethnicities other than White, and more deprived than the lower-cost (*n* = 81 194) group (all *P*-values <0.0001) (Table 1). They had an average of 4.28 LTCs (standard deviation [SD] 1.99), with chronic pain, anxiety, hypertension, depression, and osteoarthritis the most prevalent LTCs.

**Table 1.** Description of study sample, individuals with multimorbidity in 2019/2020^a^

**Characteristic**	**All**	**High primary care costs**		***P*-value**

		**Yes**	**No**	
***n***	101 498	20 304	81 194	

**Age, years, mean (SD)**	52.24 (17.98)	58.21 (18.19)	50.75 (17.61)	<0.0001

**Sex, % female**	55.87	64.33	53.75	<0.0001

**Ethnicity, %**				<0.0001
White	54.38	50.10	55.45	
Black	25.44	30.04	24.29	
Asian	6.69	7.64	6.46	
Mixed	4.70	4.68	4.71	
Other	2.41	2.65	2.35	

**IMD quintile, %^b^**				<0.0001
1 (most deprived)	23.59	26.70	22.82	
2	21.42	21.53	21.39	
3	17.48	16.84	17.64	
4	18.55	17.56	18.79	
5 (least deprived)	17.94	16.58	18.28	

**Number of LTCs, mean (SD)**	3.28 (1.6)	4.28 (1.99)	3.03 (1.38)	<0.0001

**Five most prevalent LTCs, %**				<0.0001
Chronic pain	55.46	76.52	50.19	
Anxiety	49.96	50.52	49.83	
Hypertension	33.98	45.74	31.04	
Depression	43.09	45.12	42.59	
Osteoarthritis	18.55	30.30	15.61	

**Primary care consultations, mean (SD)**	9.20 (9.47)	23.03 (11.08)	5.74 (4.66)	<0.0001

a *Percentages do not total 100% because of missing values.*

b *This is a composite index aimed at measuring social deprivation based on seven domains, including income, education, unemployment, crime, and housing, among others.^29^ IMD = Index of Multiple Deprivation. LTC = long-term condition. SD = standard deviation.*

A higher prevalence of chronic pain (76.52% versus 50.19%), hypertension (45.74% versus 31.04%), and osteoarthritis (30.30% versus 15.61%) was observed among high-versus lower-cost individuals (*P*<0.0001). The high-cost group used primary care four times more than the lower-cost group (mean annual consultation 23.03 [SD 11.08] versus 5.74 [SD 4.66], *P*<0.0001).

### Primary care costs and disease combinations

As consultation costs of high-cost individuals amounted to £13.1 million (Supplementary Table S1), this 20% of the sample incurred 53% of total consultation costs of the multimorbid population. Disproportionality starts at the 70th percentile, with costs for individuals in the 70th–80th percentile accounting for 14% of total costs (Supplementary Table S2).

The mean annual cost of consultations by high-cost individuals was £644 (SD 269) compared with £146 (SD 115) for lower-cost individuals. The cost distribution for high cost was more right-skewed and had a longer tail (Supplementary Table S1).

High-cost individuals had a total of 6833 unique combinations of LTCs, which represent 51% of all combinations in the whole sample (Supplementary Table S5) and nearly three times more heterogeneity than lower-cost individuals. The combination of LTCs with the largest contribution to costs was the trio of anxiety and chronic pain and depression (5.12%), followed by the pairs anxiety and chronic pain (2.52%), and anxiety and depression (2.37%) (Table 2). The top 10 combinations of LTCs, by share of costs, involve six LTCs: anxiety, chronic pain, depression, asthma, hypertension, and osteoarthritis. Sensitivity analyses showed similar results (Supplementary Tables S3 and S4).

**Table 2.** Top 10 LTC combinations based on their contribution to total primary care costs for high- cost individuals with multimorbidity, 2019/2020 (*n* = 20 304; total number of LTC combinations, *n* = 6833)

**LTC combination**	**Count (*n* = 20 304)**	**%**	**Primary care costs**					
			**Mean, £**	**SD, £**	**Median, £**	**IQR, £**	**Total, £**	**Total, %**
**AX&CP&DP**	1077	5.30	620.71	244.74	547.25	243	668 504.50	5.12
**AX&CP**	551	2.71	596.79	211.76	525.25	221.50	328 829.75	2.52
**AX&DP**	562	2.77	550.48	157.76	496.25	151.25	309 368.50	2.37
**AX&AT&CP&DP**	343	1.69	659.79	267.55	565.50	293	226 309.00	1.73
**CP&DP**	305	1.50	583.95	200.54	513.25	203	178 103.25	1.36
**AX&AT**	246	1.21	552.82	146.41	508.75	162	135 992.75	1.04
**CP&HY**	214	1.05	575.17	173.07	536.50	209	123 086.75	0.94
**AX&AT&CP**	175	0.86	611.81	220.09	552.50	280.75	107 066.25	0.82
**CP&HY&OA**	180	0.89	586.79	217.70	512.50	210.50	105 621.75	0.81
**AX&CP&DP&OA**	164	0.81	637.72	241.15	580.13	283.75	104 586.00	0.80

*AT = asthma. AX = anxiety. CP = chronic pain. DP = depression. HY = hypertension. IQR = interquartile range. LTC = long-term condition. OA = osteoarthritis. SD = standard deviation.*

Among the lower-cost population, the combination of LTCs with the highest contributions to costs were also the pair anxiety and depression (6.92%), the trio anxiety and chronic pain and depression (5.87%), and the pair anxiety and chronic pain (3.89%) (Table 3). Osteoarthritis did not feature in the top 10 combinations for lower-cost individuals, whereas it did with the high-cost individuals, but diabetes did feature for the lower-cost individuals.

**Table 3.** Top 10 LTC combinations based on their contribution to total primary care costs, for lower-cost individuals with multimorbidity, 2019/2020 (*n* = 81 194; total number of LTC combinations, *n* = 9354)

**LTC combination**	**Count (*n* = 81 194)**	**%**	**Primary care costs**					
			**Mean, £**	**SD, £**	**Median, £**	**IQR, £**	**Total, £**	**Total, %**
**AX&DP**	8050	9.91	101.52	104.24	78	167.00	817 227.25	6.92
**AX&CP&DP**	4688	5.77	147.83	115.46	134	189.00	693 038.75	5.87
**AX&CP**	3232	3.98	142.31	113.95	123.25	187.25	459 949.00	3.89
**AX&AT**	3191	3.93	103.55	103.51	78	165.00	330 439.50	2.80
**CP&DP**	2249	2.77	123.53	112.24	101	184.00	277 814.75	2.35
**DM&HY**	1633	2.01	143.31	99.68	128	145.25	234 033.25	1.98
**CP&HY**	1122	1.38	160.57	110.63	153.25	170.50	180 162.75	1.52
**AX&AT&CP&DP**	1116	1.37	158.23	116.36	147	186.87	176 587.50	1.49
**AX&AT&DP**	1336	1.65	123.46	111.80	102.13	187.87	164 939.75	1.40
**AT&CP**	1250	1.54	126.27	110.60	101	168.00	157 835.00	1.34

*AT = asthma. AX = anxiety. CP = chronic pain. DM = diabetes mellitus. DP = depression. HY = hypertension. IQR = interquartile range. LTC = long-term condition. SD = standard deviation.*

### LCA

Within high-cost individuals, five clusters proved the best grouping (Supplementary Table S6). No single combination of LTCs accounted for a large proportion of the clusters, except for cluster 1 where 15% of individuals had the trio anxiety and chronic pain and depression (Table 4). This cluster was younger (mean age 44 years, SD 14), with a high percentage of females (75%). For the remaining clusters, all LTC combinations had a prevalence <5%. In three of the five clusters, mental health conditions were prominent (30%, 63%, and 79% in clusters 1, 2, and 4 had depression, respectively), along with asthma (cluster 1), alcohol dependence (cluster 2), and chronic pain (cluster 4). In cluster 3, diabetes and osteoarthritis were the most prevalent LTCs (39% each). Individuals in cluster 5 showed a noticeable prevalence of cardiac diseases and chronic kidney disease, accompanied by other LTCs (average of 6.97 LTCs, SD 1.94), and had the highest average costs (£725, SD 336). They also had the highest average age (78.64 years, SD 11.37) and lowest proportion from deprived areas (24.47%).

**Table 4.** Results of LCA: clusters among high-cost individuals with multimorbidity, 2019/2020 (*n* = 20 304)

**Item**	**Cluster 1**	**Cluster 2**	**Cluster 3**	**Cluster 4**	**Cluster 5**
***n***	7283	1566	7107	2198	2150
**%**	35.87	7.71	35.00	10.83	10.59
**Contribution to primary care costs, %**	34.56	8.29	33.26	11.96	11.94
**Primary care consultations, mean (SD)**	21.46 (9.97)	24.93 (12.46)	22.04 (9.73)	26.09 (12.67)	27.11 (13.85)
**Primary care costs, £, mean (SD)**	620.06 (253.69)	691.67 (300.99)	611.44 (227.53)	711.02 (306.83)	725.46 (335.65)
**Primary care costs, £, median**	542.50	596.50	541.00	619.75	627.75
**Primary care costs, £, IQR**	233.75	314.00	225.25	336.00	337.75
**Unique LTC combinations, total *n***	661	1002	2097	1257	1816
**LTCs, mean (SD)**	3.16 (1.05)	5.12 (1.86)	3.68 (1.34)	6.74 (1.49)	6.97 (1.94)
**Mostly prevalent individual LTCs, %**	DP, 30; AT, 29	DP, 63; AX, 48; AD, 47	DM, 39; OA, 39	CP, 100; DP, 79	CHD, 50; CKD, 49; DM, 48; HF, 46
**Most prevalent LTC combinations, %**	AX&CP&DP, 15; AX&DP, 8; AX&CP, 8; AX&AT&CP&DP, 5	All combinations <5% prevalence	All combinations <5% prevalence	All combinations <5% prevalence	All combinations <5% prevalence
**Dead, during study year, %**	2.87	3.58	2.69	3.23	2.70
**Age, years, mean (SD)**	43.97 (13.93)	53.22 (12.03)	65.21 (14.85)	66.32 (12.18)	78.64 (11.37)
**Sex, % female**	75.00	39.85	60.17	72.00	51.30
**IMD, % decile 1 (most deprived)**	26.27	29.25	26.73	28.43	24.47
**Ethnicity, % White**	54.74	66.60	39.81	52.96	53.49

*AD = alcohol dependence. AT = asthma. AX = anxiety. CHD = coronary heart disease. CKD = chronic kidney disease. CP = chronic pain. DP = depression. HF = heart failure. IMD = Index of Multiple Deprivation (locally derived). IQR = interquartile range. LCA = latent class analysis. LTC = long-term condition. OA = osteoarthritis. SD = standard deviation.*

In four of the five clusters, the average count of LTCs was higher than the average of each of the lower-cost clusters. Clusters 2 and 4 had the highest rates of patients dying (3.58% and 3.23%, respectively); this was higher than any of the lower-cost clusters (Tables 4 and 5).

**Table 5.** Results of LCA: clusters among low-cost individuals with multimorbidity, 2019/2020 (*n* = 81 194)

**Item**	**Cluster 1**	**Cluster 2**	**Cluster 3**
***n***	29 415	16 126	35 653
**%**	36.23	19.86	43.91
**Contribution to primary care costs, %**	43.09	18.06	38.85
**Primary care consultations, mean (SD)**	7.21 (4.74)	5.25 (4.69)	4.76(4.26)
**Primary care costs, £, mean (SD)**	173.09 (112.19)	132.31 (114.54)	128.77 (113.61)
**Primary care costs, £, median**	165.75	113.50	108.00
**Primary care costs, £, IQR**	183.75	187.75	186.00
**Unique LTC combinations, total *n***	6027	2827	500
**LTCs, mean (SD)**	3.57 (1.70)	2.90 (1.31)	2.64 (0.87)
**Mostly prevalent individual LTCs, %**	CP, 45; DM, 39	CP, 49; AT, 40	CP, 46; DP, 31
**Most prevalent LTC combinations, %**	DM&HY, 6	AT&CP, 8	AX&DP, 23; AX&CP&DP, 13; AX&CP, 9; AX&AT, 9; CP&DP, 6
**Dead, during study year, %**	2.90	2.86	2.89
**Age, years, mean (SD)**	65.43 (13.87)	46.64 (14.35)	40.49 (12.79)
**Sex, % female**	50.63	43.80	60.83
**IMD, % decile 1 (most deprived)**	24.73	24.47	20.49
**Ethnicity, % White**	43.04	55.69	65.59

*AD = alcohol dependence. AT = asthma. AX = anxiety. CP = chronic pain. DM = diabetes mellitus. DP = depression. HY = hypertension. IMD = Index of Multiple Deprivation (locally derived). IQR = interquartile range. LCA = latent class analysis. LTC = long-term condition. SD = standard deviation.*

Three clusters best grouped lower-cost individuals (Supplementary Table S7). All three were characterised by a relatively high prevalence of chronic pain (45%, 49%, and 46% across clusters 1, 2, and 3, respectively), along with diabetes (cluster 1), asthma (cluster 2), and depression (cluster 3) (Table 5). Unlike the high-cost group, each cluster had at least one combination of LTCs with >5% prevalence (for example, 6% in cluster 1 had diabetes and hypertension, and 23% in cluster 3 had anxiety and depression). Cluster 1 had the highest average cost at £173.09 (SD 112.19), the highest average age (65.43 years, SD 13.87), and lowest percentage of White ethnicity (43.04%).

## Discussion

### Summary

High-cost individuals with multimorbidity accounted for 53% of the total costs of primary care consultations. They were grouped into five clusters, with mental health (anxiety and depression) highly prevalent in three clusters. People with chronic pain were concentrated in one cluster, with a prevalence in the whole high-cost sample of 77%. Individuals with cardiac conditions dominated another cluster and alcohol dependence (along with depression and anxiety), asthma, and osteoarthritis were also present across clusters.

High-cost individuals are a more heterogeneous group of patients than those with lower costs. This is evidenced by: the higher mean number of LTCs (4.28 versus 3.03, *P*<0.0001), the larger number of clusters (five versus three), and the three times larger number of LTC combinations. High-cost individuals only showed combinations of LTCs >5% prevalence in one of five clusters, meaning no single combination of LTCs made a sizeable contribution to primary care costs, the largest being anxiety, chronic pain, and depression. This contrasts with the lower-cost group where each cluster had between one and five LTC combinations >5%, indicating a greater concentration in specific combinations of LTCs.

### Strengths and limitations

This study advances existing literature on health service use and costs relating to individuals with multimorbidity by focusing on the patients with the most expensive cases. Descriptive analyses of combinations of LTCs and their contribution to costs are complemented by latent class analyses to understand clusters of individuals with similar patterns of LTCs. A large sample with urban, deprived, and ethnically diverse individuals was used, with sociodemographic, medical, and primary care use information. This fills an important research gap given the crucial role of primary care in managing an increasingly multimorbid population. The diverse population allows for meaningful analysis of ethnic minority groups, who are typically under-represented in health research.^30^ Costs reflect the workload of primary care providers. Identifying high-workload individuals is important given the current mismatch between workforce demand and supply.

Results may not be generalisable to rural, older, or less ethnically diverse populations. For example, having a smaller proportion of patients from ethnic minority groups may reduce prevalence of hypertension and diabetes,^31^^,^^32^ whereas older populations may have more multimorbidity given its correlation with age.^33^

Primary care costs reflect consultations only and the inclusion of medication costs may affect results since the cost of medication relative to total costs can vary by condition.^34^^–^36 Averages from the Personal Social Services Research Unit were used for consultation duration. Actual duration may vary by LTC. LTCs were defined based on diagnostic Read codes that may miss patients who do not have a formal diagnosis despite receiving treatment, the extent of which may vary by LTC.^37^ Similarly, the presence of a condition was defined as a diagnostic code before or during the study period and, therefore, any changes to diagnoses were not considered. Limited by cross-sectional data, the current study could not differentiate between 1-year high-cost individuals and people with more persistent conditions over time.^25^ The current study considers primary care only, and it is not clear whether conclusions would hold across secondary and social care settings, or the extent to which the degree of substitution and complementarity among care components should be accounted for.^13^ Finally, the current study describes costs only, and this debate should also be informed by outcomes.

### Comparison with existing literature

Previous literature on clustering multimorbid individuals has not focused on the high-cost group, yet some similarities are apparent. Zhu *et al* identified the highest primary care use in clusters with depression, anxiety, and pain.^18^ These LTCs were highly prevalent among the clusters found in the current study. Soley-Bori *et al* pointed at the alcohol dependence and substance dependence cluster as the one with the highest expected primary care demand as further LTCs develop.^22^ In the current study, alcohol dependence was common in one of the clusters with high-cost individuals. Stokes *et al* concluded that no single combination of LTCs contributed significantly to total secondary care costs, with the pair diabetes and hypertension representing the highest share (3.2%).^21^ The current study reached similar conclusions based on primary care data. A total of 13 388 LTC combinations existed in the overall multimorbid sample, 6833 among the high-cost subgroup. No single combination made a major contribution to primary care costs, with anxiety, chronic pain, and depression accounting for just 5% of total primary care costs incurred by high-cost-multimorbid individuals.

Tran *et al* found the combination of cancer and mental health conditions was the most expensive.^38^ The current study did not find cancer to be a major contributor to primary care costs. However, Tran *et al* included hospital admissions and outpatient care, suggesting cancer incurs significant costs outside primary care compared with other LTCs.^38^

### Implications for research and practice

Enhancing care coordination across specialties caring for individuals with multimorbidity is considered necessary for high-quality and efficient care.^39^ This strategy is particularly important for high-cost individuals, who use a disproportionate percentage of healthcare services. Understanding the most common clusters of LTCs may help to prioritise care coordination and integration efforts.^2^ Three of the five ‘high-cost’ clusters identified in this study had a high prevalence of anxiety and depression, suggesting mental health issues expand primary care use in this group. Prioritising mental health and enhancing its coordination with physical LTCs may improve outcomes and reduce costs among high-cost individuals. The Improving Access to Psychological Therapies for people with LTCs (called IAPT-LTC), currently underway, is an example of an initiative to facilitate access to mental health services and coordination between mental and physical health providers.^40^ Further research on the impact of mental health on healthcare needs, costs, and outcomes of individuals with multimorbidity is needed.

Findings from this study underscore the wide heterogeneity of high-cost individuals with multimorbidity and further research should consider if this is associated with increased clinical complexity and a higher risk of unmet need and/or poorer health outcomes. Individualised care, tailored and centred around each patient, reflecting their preferences — rather than one-size-fits-all strategies — seems like the appropriate response. This conflicts with limited primary care resources and mostly single-disease-oriented payment incentives such as the Quality and Outcomes Framework. To ease this tension, research is needed on enhanced payment mechanisms that transcend disease boundaries and explore the design of cost-effective interventions that facilitate — through technology, data, or other mechanisms — healthcare delivery for individuals with multimorbidity.

## Data

The datasets generated and/or analysed during the current study are not publicly available because the condition for pseudonymised data extraction by included general practices was that the data would not be shared beyond parties named in the data-sharing agreement, but are available from the corresponding author on reasonable request.

## References

[1] Souza DLB, Oliveras-Fabregas A, Minobes-Molina E (2021). Trends of multimorbidity in 15 European countries: a population-based study in community-dwelling adults aged 50 and over. BMC Public Health.

[2] Whitty CJM, MacEwen C, Goddard A (2020). Rising to the challenge of multimorbidity. BMJ.

[3] Pearson-Stuttard J, Ezzati M, Gregg EW (2019). Multimorbidity — a defining challenge for health systems. Lancet Public Health.

[4] Rijken M, Struckmann V, van der Heide I (2017). How to improve care for people with multimorbidity in Europe?.

[5] Kingston A, Robinson L, Booth H (2018). Projections of multi-morbidity in the older population in England to 2035: estimates from the Population Ageing and Care Simulation (PACSim) model. Age Ageing.

[6] Cassell A, Edwards D, Harshfield A (2018). The epidemiology of multimorbidity in primary care: a retrospective cohort study. Br J Gen Pract.

[7] Starfield B, Shi L, Macinko J (2005). Contribution of primary care to health systems and health. Milbank Q.

[8] World Health Organization (2016). Multimorbidity.

[9] Salisbury H (2021). When did you last see your doctor?. BMJ.

[10] British Medical Association (2024). Pressures in general practice data analysis. https://www.bma.org.uk/advice-and-support/nhs-delivery-and-workforce/pressures/pressures-in-general-practice-data-analysis.

[11] Haggerty JL, Reid RJ, Freeman GK (2003). Continuity of care: a multidisciplinary review. BMJ.

[12] Barnett K, Mercer SW, Norbury M (2012). Epidemiology of multimorbidity and implications for health care, research, and medical education: a cross-sectional study. Lancet.

[13] Dreyer K, Parry W, Jayatunga W, Deeny S (2019). A descriptive analysis of health care use by high-cost, high-need patients in England.

[14] Wammes JJG, van der Wees PJ, Tanke MAC (2018). Systematic review of high-cost patients’ characteristics and healthcare utilisation. BMJ Open.

[15] Blumenthal D, Chernof B, Fulmer T (2016). Caring for high-need, high-cost patients — an urgent priority. N Engl J Med.

[16] Zulman DM, Chee CP, Wagner TH (2015). Multimorbidity and healthcare utilisation among high-cost patients in the US Veterans Affairs Health Care System. BMJ Open.

[17] Bisquera A, Gulliford M, Dodhia H (2021). Identifying longitudinal clusters of multimorbidity in an urban setting: a population-based cross-sectional study. Lancet Reg Health Eur.

[18] Zhu Y, Edwards D, Mant J (2020). Characteristics, service use and mortality of clusters of multimorbid patients in England: a population-based study. BMC Med.

[19] Hernández B, Voll S, Lewis NA (2021). Comparisons of disease cluster patterns, prevalence and health factors in the USA, Canada, England and Ireland. BMC Public Health.

[20] Zemedikun DT, Gray LJ, Khunti K (2018). Patterns of multimorbidity in middle-aged and older adults: an analysis of the UK Biobank Data. Mayo Clin Proc.

[21] Stokes J, Guthrie B, Mercer SW (2021). Multimorbidity combinations, costs of hospital care and potentially preventable emergency admissions in England: a cohort study. PLoS Med.

[22] Soley-Bori M, Bisquera A, Ashworth M (2022). Identifying multimorbidity clusters with the highest primary care use: 15 years of evidence from a multi-ethnic metropolitan population. Br J Gen Pract.

[23] French E, Kelly E (2016). Medical spending around the developed world. Fisc Stud.

[24] Curtis L, Burns A (2021). Unit costs of health and social care 2020. https://www.pssru.ac.uk/project-pages/unit-costs/unit-costs-2020.

[25] Chang HY, Boyd CM, Leff B (2016). Identifying consistent high-cost users in a health plan. Med Care.

[26] Garfinkel SA, Riley GF, Iannacchione VG (1988). High-cost users of medical care. Health Care Financ Rev.

[27] Kuha J (2004). AIC and BIC: comparisons of assumptions and performance. Sociol Methods Res.

[28] Celeux G, Soromenho G (1996). An entropy criterion for assessing the number of clusters in a mixture model. J Classif.

[29] Ministry of Housing, Communities and Local Government (2019). English indices of deprivation 2019. https://www.gov.uk/government/statistics/english-indices-of-deprivation-2019.

[30] Redwood S, Gill PS (2013). Under-representation of minority ethnic groups in research — call for action. Br J Gen Pract.

[31] Lane D, Beevers DG, Lip GYH (2002). Ethnic differences in blood pressure and the prevalence of hypertension in England. J Hum Hypertens.

[32] Pham TM, Carpenter JR, Morris TP (2019). Ethnic differences in the prevalence of type 2 diabetes diagnoses in the UK: cross-sectional analysis of the Health Improvement Network primary care database. Clin Epidemiol.

[33] Ledwaba-Chapman L, Bisquera A, Gulliford M (2021). Applying resolved and remission codes reduced prevalence of multimorbidity in an urban multi-ethnic population. J Clin Epidemiol.

[34] Heslin M, Babalola O, Ibrahim F (2018). A comparison of different approaches for costing medication use in an economic evaluation. Value Health.

[35] Dagenais S, Caro J, Haldeman S (2008). A systematic review of low back pain cost of illness studies in the United States and internationally. Spine J.

[36] Liu JL, Maniadakis N, Gray A, Rayner M (2002). The economic burden of coronary heart disease in the UK. Heart.

[37] Archer C, Turner K, Kessler D (2022). Trends in the recording of anxiety in UK primary care: a multi-method approach. Soc Psychiatry Psychiatr Epidemiol.

[38] Tran PB, Kazibwe J, Nikolaidis GF (2022). Costs of multimorbidity: a systematic review and meta-analyses. BMC Med.

[39] Smith SM, Soubhi H, Fortin M (2012). Interventions for improving outcomes in patients with multimorbidity in primary care and community settings. Cochrane Database Syst Rev.

[40] NHS England (2018). The Improving Access to Psychological Therapies (IAPT) pathway for people with long-term physical health conditions and medically unexplained symptoms. https://www.england.nhs.uk/publication/the-improving-access-to-psychological-therapies-iapt-pathway-for-people-with-long-term-physical-health-conditions-and-medically-unexplained-symptoms.

